# Multi-Component Herbal Products in the Prevention and Treatment of Chemotherapy-Associated Toxicity and Side Effects: A Review on Experimental and Clinical Evidences

**DOI:** 10.3389/fphar.2018.01394

**Published:** 2018-11-29

**Authors:** Bowen Fu, Ning Wang, Hor-Yue Tan, Sha Li, Fan Cheung, Yibin Feng

**Affiliations:** School of Chinese Medicine, Li Ka Shing Faculty of Medicine, The University of Hong Kong, Hong Kong, China

**Keywords:** multi-component, herbal products, cancer chemotherapy, side effects, experimental study, clinical trials

## Abstract

Chemotherapy is nowadays the main treatment of human cancers. Chemotherapeutic agents target rapidly dividing cancer cells to suppress tumor progression, however, their non-specific cytotoxicity often leads to significant side effects that might be intolerable to cancer patients. Multi-component herbal products have been used for thousands of years for the treatment of multiple human diseases. This study aims to systematically summarize and evaluate the experimental and clinical evidences of the efficacy of multi-component herbal products in improving chemotherapy-induced side effect. Literature was retrieved from PubMed database and evaluated based on the side effects described. Multi-component herbal products were found to be effective in ameliorating the neurotoxicity, gastrointestinal toxicity, hematological toxicity, cardiotoxicity, hepatotoxicity and nephrotoxicity. Both experimental and clinical evidences were found, indicating the potential of applying multicomponent herbal products in the clinical treatment of chemotherapy-induced side effects. However, the lack of mechanistic and pharmacokinetic studies, inconsistency in product quality, as well as insufficient clinical evidence suggested that more investigations are urgently necessary. In all, our review shed light on the potential of using multi-component herbal products in the clinical management of chemotherapy-induced toxicity and side effects. We also discussed the potential threats of natural products for cancer treatment and compared the advantages of using herbs to conventional chemical drugs.

## Introduction

Cancer remains to be the leading cause of human death all over the world. Observing the remarkable progress of development of new treatment against human cancer, the survival rate and quality of life (QOL) of cancer patients have been largely improved over the last decades (Courneya and Friedenreich, 1999). Chemotherapy is one of the major therapeutic approaches, which enables prolonged life span and reduced cancer progression. Chemotherapy targets on rapid-dividing cancer cell to suppress its growth or promote major endocrinal signals of, which is called hormonal therapies (Steinmeyer, 1999). However, due to the low specificity of chemotherapeutic agents, its adverse reaction and toxicity are commonly observed during chemotherapeutic treatment to cancer patients, which largely affect its efficacy and application as patients are normally intolerable to the side effects caused by chemotherapeutic agents (Allemani and Coleman, 2017). Intolerance in cancer patients therefore results in reduction of chemotherapy dose or even discontinuation of the treatment (Molina et al., 2008). Common side effects include neurotoxicity, diarrhea, nausea, emesis, myelosuppression and anorexia, etc. In this case, development of an appropriate way to alleviate the side effects of chemotherapy is still necessary and urgent (Andersen, 1992).

Natural products are chemicals produced in nature by living organisms, which usually possess some particular pharmacological or biological activities. A great number of natural products are from medicinal plants, which have been used to treat human diseases for thousands of years in Asia (Koehn and Carter, 2005). Mounting evidence have suggested that the major events and signaling pathways in human cancers can be potentially modulated by natural products and novel herbal compounds (Ko and Auyeung, 2013). In particular, recent-year evidence from studies on cell line, animal and clinical trials have revealed that a large number of natural products were potentially active in ameliorating chemotherapy-induced side effects. Compounds and single herb extracts and composite herbal formula were reported to enhance the QOL of patients with cancers. As the effect of pure natural compounds have been largely reviewed elsewhere. In this review, we therefore only focused on multi-component herbal extracts. We retrieved recent studies from Pubmed database, and summarized the most common side effects caused by first-line chemotherapy, which were alleviated by multi-component herbal extracts. Major mechanisms of action in reducing chemotherapy-induced side effects were also discussed.

## Chemotherapy-Induced Side Effects Reduced by Natural Products

Classical chemotherapeutic agents are cytotoxic by interfering cell division (mitosis). Compared with normal cells, cancer cells are supposed to be more sensitive to chemotherapy because of its rapid-dividing capability. However, non-specific cytotoxic effects of chemotherapeutic agents are usually observed in different tissues and organs of human body where some particular cell types physiologically divide rapidly. These toxic reactions include nephrotoxicity, neurotoxicity, cardiotoxicity, hematological toxicities, gastrointestinal toxicity and hepatotoxicity (Early Breast Cancer Trialists’ Collaborative Group (EBCTCG), 2005). Natural products, including pure compounds, herb extracts and composite formula, have been reported to effectively reduce the side effects induced by chemotherapy (Chen et al., 2010) and maintain the QOL of cancer patients. At present, non-specific toxicity of first-line chemotherapeutic agents such as 5-Fluorouracil (5-Fu), capecitabine and oxaliplatin have been largely reported.

## Neurotoxicity

Neurotoxicity is caused by platinum-based chemotherapeutic agents such as cisplatin and oxaliplatin, which can damage the nervous system and result in many symptoms including weakness or numbness, loss of memory and delusions (Berger et al., 1997). The side effects can be acute or chronic for several days, they are reversible and its chronic symptoms reported to continue up to 4 years (du Bois et al., 1999). Symptoms may express in patients’ hands, feet, the tissues around the mouth such as pharynx and larynx, and paresthesia, which include numbness, nerves tingling, poking, pruritus, or sensations of burning. The pain caused by muscle weakness and hypoesthesia is also included.

*Cortex Phellodendron* chinensis (CPC) as well as *Cortex Phellodendron* amurensis (CPA) are descendent from the dried bark of *Phellodendron chinense* Schneid. or *P. amurense* Rupr., respectively. A cell study using P12 cell lines with CPC and CPA treated, the western blotting and statistical analysis had been done. The results showed that CPC and CPA can reduce the neurotoxicity by elevating the ratio of the mRNA and protein levels of Bcl-2/Bax and expression of caspase-3 (Xian et al., 2013). Goshajinkigan, a compound formula comprised of ten herbs, is usually used in Japan to treat numbness or pain. It is reported that cold hypersensitivity induced by oxaliplatin was associated with transient receptor potential melastatin 8 (TRPM8) and transient receptor potential Ankyrin 1 (TRPA1) channels, which are ion channels that are activated by cold (Kawashiri et al., 2012). Animal studies have proved that Goshajinkigan prevented acute peripheral neuropathy induced by oxaliplatin but not affect its treating cancer effect (Kuriyama and Endo, 2018) by inhibiting functional variations of the transient receptor potential (TRP) channels, which effect mainly TRPA1 and TRPM8 (Kato et al., 2014). A reminiscent study and a randomized clinical trial showed that Goshajinkigan significantly reduced the incidence of neurotoxicity, prolonged the treatment time of oxaliplatin and delayed the occurrence of neurotoxicity (Oki et al., 2015). A clinical study was involved in patients who were suffered advanced or recrudescent colorectal cancer received standard FOLFOX regimens. It was divided in treatment group and placebo-controlled group, which was a double-blinded phase II study. From the result, goshajinkigan at receivable safety range showed hopeful impression to delay the neurotoxicity whose grade is equal or greater than two (Kono et al., 2013).

Keishikajutsubutou is a compound formula composed by cassia twig, monkshood and rhizome atractylodis, which is usually used to treat arthralgia and neuralgia. A clinical study showed that Keishikajutsubutou (TJ-18) with powdered processed aconite root (TJ-3023) could reduce the peripheral neuropathy induced by oxaliplatin in patients with colon cancer. Among 11 patients with colon cancer used TJ-18 (7.5 g) and TJ-3023 (2 g) combined with FOLFOX6 and FOLFOX7, warm feelings in feet and hands were occurred in 6 patients, and 5 patients was observed reduction in neuropathy (Yamada et al., 2012).

From the reviews, it is reported that the efficacy of traditional Chinese Medicines in preventing chemotherapy-induced neurotoxicity, randomized controlled trials (RCTs) that evaluated the efficacy of TCMs in preventing chemotherapy-induced neurotoxicity in cancer patients were included. Studies were searched in PubMed, EMbase, CNKI, Wan Fang and Wei Pu database. Twenty-five RCTs (1572 patients) involving five TCMs were included. From the results, compared with oxaliplatin-based chemotherapy alone, the combination with Goshajinkigan, Keishikajutsubutou, HuangQi Injection (SMI), Shenfu Injection (SFI), Buyang Huanwu Decoction (BHD), and Huangqi Guizhi Wuwu Decoction (HGWD) could decrease the overall and severe CIPN incidence in cancer patients (Table 1).

**Table 1:** Clinical trials with TCM combined with chemotherapy to affect the CIPN.

Number of patients		Type of cancer	CT regimen	Type of TCM	Number of Ct cycle by assessment	Outcomes	Modified Jadad scores	Reference
							
Treatment	Control							
30	30	CRC	FOLFOX	HQI	3	➀➁	3	(Chen, 2009)
20	20	GIC	FLOFOX	HQI	1	➀➁	4	(Cui et al., 2017)
47	46	GIC	OAX+RAL	HQI	3	➀➁	5	(Chen and Wang, 2017)
46	50	GIC	FLOFOX	SMI	4	➀➁	3	(Fang et al., 2012)
30	30	GIC	OXA-based regimen	SMI	4	➀➁	4	(Gao and Dai, 2012)
46	41	CRC	FOLFOX	SMI	4	➀➁	3	(Zhu et al., 2013)
46	41	CRC	FLOFOX	SMI	4	➀➁	3	(Zhang et al., 2013)
46	45	GC	FLOFOX	SMI	4	➀	3	(Zhong and Zang, 2014)
30	30	GIC	FLOFOX	SFI	4	➀➁	3	(Jorden, 2012)
40	40	GIC	FLOFOX	SFI	3	➀➁	4	(Zhu et al., 2010)
32	32	GIC	FLOFOX	SFI	3	➀➁	4	(Zhang et al., 2014)
21	21	CRC	FLOFOX	SFI	4	➀	3	(Yao et al., 2009)
37	36	CRC	FLOFOX	SFI	4	➀➁	3	(Zhang et al., 2006)
20	20	CRC	FLOFOX	BHD	6	➀➁	3	(Wu et al., 2012)
30	30	GIC	FLOFOX	BHD	4	➀➁	4	(Wen and Zhen, 2010)
19	18	NS	FLOFOX	BHD	3	➀	3	(Yang et al., 2007)
20	20	GIC	FLOFOX	BHD	4	➀➁	3	(Yang, 2011)
35	35	CC	FLOFOX	BHD	3	➀➁	4	(Bao and Cao, 2011)
21	21	NS	FLOFOX	HGWD	4	➀➁	3	(Zou and Zhang, 2015)
24	24	GIC	FLOFOX	HGWD	4	➀➁	4	(Lu et al., 2010)
30	30	CC	FLOFOX	HGWD	4	➀➁	3	(Wang, 2015)
30	30	CRC	FLOFOX	HGWD	4	➀➁	4	(Li et al., 2014)
20	20	CRC	FLOFOX	HGWD	3	➀➁	4	(Grothey, 2005)
45	45	GIC	FLOFOX	HGWD	2	➀➁	3	(Yuan, 2012)
40	40	GIC	FLOFOX	HGWD	8	➀➁	4	(Lin and Liqun, 2011)
31	31	GIC	FLOFOX	HGWD	4	➀➁	3	(Ya, 2011)


*➀Severe CIPN incidence; ➁overall CIPN incidence; CT, chemotherapy; GIC, gastrointestinal cancer; CRC, colorectal cancer; CC, colon cancer; GC, gastric cancer; OXA, oxaliplatin; RAL, raltitrexed; NS, not stated; FOLFOX, Oxaliplatin+Fluorouracil+Leucovorin; XELOX, Capecitabine+Oxaliplatin; HQI, Huangqi injection; SMI, Shenmai injection; SFI, Shenfu injection; BHD, Buyang Huanwu Decoction; HGWD, Huangqi Guizhi Wuwu Decoction.*

## Gastrointestinal Toxicity

Irinotecan (CPT-11, Camptosar) is used in combination with 5-fluorouracil (5-fu) and leucovorin (LV) as the first-line of treatment for metastatic colon or rectal carcinoma. CPT-11 is also approved for second-line treatment of recurrent metastatic colon or rectal carcinoma after 5-FU-based therapy. The side effects of CPT-11 include vomiting, nausea, and diarrhea, which can be serious. Colonic ulceration caused by intestinal cell death and inflammation, often accompanied by gastrointestinal bleeding, has been observed in combination with CPT-11. Recent years, TCM has been used to decrease the side effects of gastrointestinal toxicity induced by chemotherapy. In PubMed, 75 clinical trial publications were searched for the key words herbal TCM and cancer. However, only five clinical trials maintain a clinically relevant endpoint. The Cochrane Library did not contain a review or clinical trial that describe clinical efficacy of herbs in cancer patients.

**FIGURE 1:**
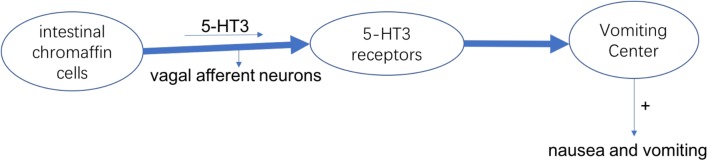
The mechanism of nausea and vomiting induced by 5-HT3.

The most common symptom in chemotherapy-induced gastrointestinal toxicity is chemotherapy-induced nausea and vomiting (CINV), which can make reduction of acceptance of chemotherapy, intension of organ functions and degradation in QOL. The cause of nausea and vomiting is the activation of vagal afferent neurons. In intestinal chromaffin cells, they can release 5-Hydroxytryptamine-3 (5-HT3) and substance P by binding to 5-HT3 as well as neurokinin (NK)-1 receptors, leading to nausea and vomiting (Watcha and White, 1992). At present, it has been developed to use medicines that can against 5-HT3 and NK-1 receptors to treat CINV (Figure 1; Rojas et al., 2014). From the international guidelines of antiemetic, patients who receive chemotherapy such as cisplatin are recommended to use the antagonists of 5-HT3 and NK-1 receptors and corticosteroids. However, this treatment effect does not apply to all patients, because the complete response (CR) and control rate were 30–90% from the report, and about 15% of patients were reported to have anorexia, which is considered as an adverse event (Navari, 2017). In addition, after receiving chemotherapy 7 days, the food intake of patients decreased to 25% of baseline (Francois et al., 2016).

**FIGURE 2:**
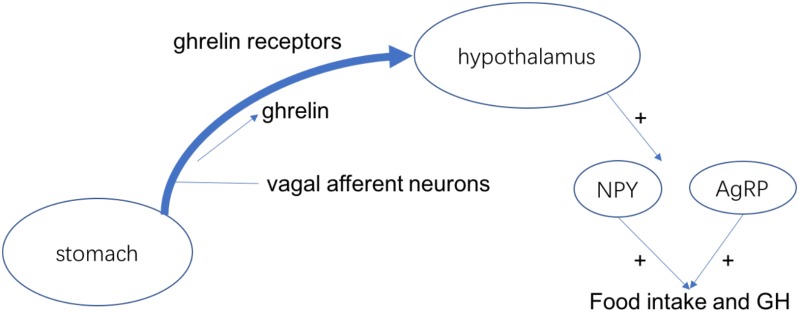
The mechanism of ghrelin regulates the food intake and GH.

Ghrelin is a kind of peptide hormone produced by ghrelin cells in the gastrointestinal tract, which plays the role of neuropeptide in the central nervous system. In addition to regulating appetite, ghrelin also plays an important role in adjusting the distribution as well as use speed of energy (Takeda et al., 2012). Ghrelin has 28 amino acids and plays miscellaneous physiological roles as a current hormone, for example, it can induce growth hormone release and food intake (Vaudry et al., 2000). For the rodents, ghrelin can regulate the food intake and weight gain in the venae. The ghrelin receptor is considered to active the NPY/AgRP neurons to stimulate food intake because it is expressed in the food intake-stimulating neurons of hypothalamus (Schele et al., 2016). Stomach secrets the ghrelin and vagal afferent neurons produce the ghrelin receptors. The interaction makes them bind and the combinations are transported to the terminus of the afferent fibers, which can inhibit the electrical activity of the vagal afferent fibers and control the food intake and GH secretion (Figure 2; Cowley et al., 2003). Recent studies confirmed that the level of ghrelin in the blood is decreased after chemotherapy such as oxaliplatin in animals as well as humans. Therefore, ghrelin may be related in chemotherapy-induced anorexia (Shiomi et al., 2018).

Rikkunshito is a kind of compound formulas, which is constituted of 8 herbal medicines. In Japan, it is widely used to treat all kinds of gastrointestinal confusions (Harada et al., 2017). Animal experiments demonstrated that cisplatin treatment could decrease serum ghrelin levels and food intake, which can be recovered by antagonists of the 5-HT2B receptors (Takeda et al., 2013). Rikkunshito improved the decrease of ghrelin levels in serum and food intake induced by cisplatin. Detail analysis showed that the flavonoids rikkunshito had an opposed effect on the 5-HT2B receptors, which can recover the decreased expression of excretion of ghrelin by cisplain in the hypothalamus (Takeda et al., 2008). In addition, the 5-HT3 receptor is also been antagonized by rikkunshito from the report (Yakabi et al., 2010).

Several clinical studies have been confirmed that Rikkunshito can prevent CINV and anorexia (Takeda et al., 2012). A cross-over clinical trial involving 120 patients with unrespectable lung cancer who received treatment of highly emetogenic chemotherapy (HEC) and moderately emetogenic chemotherapy (MEC) showed that during the chemotherapy, the reduction of plasma acyl ghrelin levels and food intake as well as chemotherapy-induced anorexia were improved by rikkunshito (Harada et al., 2017). Another clinical study with patients with carcinoma of uterine cervix or corpus cancer who received cisplatin (50 mg/m (2) day 1) combined with paclitaxel (135 mg/m (2) day 0) as first-line chemotherapy revealed that Rikkunshito provided observable effect on the prevention of CINV and anorexia (Ohnishi et al., 2017).

In Korea, China and Japan, Sip-jeon-dea-bo-tang (SJDBT), a traditional herbal medicine, is widely used to treat a number of diseases, including anorexia, sickle cell disease, extreme tired and weakness (Choi et al., 2014). In a chemotherapy-induced anorexia mouse model, oral administration of SJDBT inhibited the decrease of food intake and body weight loss in chemotherapeutic agents-treated animal. The results of multiple experiments showed that through the SJDBT, the serum IL-6 level was increased, while the leptin level was controlled within normal range. Furthermore, SJDBT can active the JAK1/STAT3 signaling pathway, which leads to the increase of leptin and IL-6 levels in the fat tissue (Woo et al., 2016).

Ginger (*Zingiber officinale*),as a Chinese medicine, has been used to treat gastrointestinal diseases for hundreds of years (Ben-Arye et al., 2017). Its use in this setting was justified by its chemical properties. There is a series of bioactive compounds in the stem of ginger such as zingerone, shogaols, paradol and zingiberene. From the report, the oral and gastric secretions may be stimulated by ginger (Boon and Wong, 2004). The regulation of gastrointestinal motility and the interaction with 5HT3 and NK1 receptors involved in the CINV reflex are also the functions of ginger. What’s more, ginger has a scavenger effect on free radicals (Bossi et al., 2016).

A systematic review reported seven clinical trials of ginger with placebo or current antiemetic treatment in patients receiving chemotherapy. These patients received different chemotherapy regimens, ranging in size from 36 to 576. CINV symptoms can be assessed from 3 days before chemotherapy to 10 days after treatment. In most cases, ginger is provided as a capsule powder or standard extract based on gingerol content. Dosage was 1–2 g per day over 1–10 days. Overall, three trials have shown that the advantage of ginger in the treating acute or delayed CINV, the results of two expressed an effect similar to metoclopramide, and the rest of two had unsatisfactory results (Marx et al., 2017).

6-gingerol is a major active substance extracted from ginger. A phase II randomized double-blinded placebo-controlled study of 6-gingerol confirmed that 6-gingerol significantly improved overall CR rate in CINV, appetite and QOL in cancer patients receiving adjuvant chemotherapy. 88 patients were randomized to receive 6-gingerol 10 mg or placebo. The frequency is twice a day in 12 weeks. All of them received emetogenic adjuvant chemotherapy in a range from low to high. Compared with placebo group, the CR rate of patients in gingerol group was obviously higher (Konmun et al., 2017).

*Scutellaria barbata* D. Don is one of the mostly studied medicinal plants in the field of antitumor medicine. An animal study confirmed that that *S. barbata* has good antitumor activity and can inhibit many kinds of tumor cells, such as liver cancer and lung cancer cells (Xu et al., 2015). A study showed that 12 g/kg/day *S. barbata* extract (ESB) had inhibitory effect on tumor growth, and can improve the anticancer response and alleviating side effects of 5-FU. Though low dose (3 g/(kg/day) of ESB could not increase the inhibitory rate of 5-FU, it could significantly reduce the toxicity of chemotherapy (Dai et al., 2008).

KLT anglaite injection is one of Chinese herb preparations with confirmed anti-tumor activity (Lu et al., 2008). It is mainly used in the treatment of non-small cell lung cancer, liver cancer, gastric cancer and other tumors. KLT is a unique plant-derived molecular target agent as a micro-emulsion for intravenous use. Many clinical studies showed that combined with chemotherapy, KLT could improve the short-term efficacy, clinical performance and reduced the risk of gastrointestinal reaction compared with using systematic chemotherapy alone (Liu et al., 2014).

Jinlong capsule is a compound formula containing *Gekko japonicus*, *Hedyotis diffusa* Willd, *Agkistrodon acutus* (Guenther), which is used in combination of chemotherapy to treat non-small cell lung cancer (Li et al., 2013). A meta-analysis shows that Jinlong capsule can improve the curative effect and life quality, and decrease the gastrointestinal reaction of patients. The result of meta-analysis showed that compared with chemo-radiotherapy using alone group, combination of Jinlong capsule with chemotherapy could not only improve the patients’ curative effect, clinical benefit rate, life quality improvement rate, and decrease leucopenia incidence rate and gastrointestinal reaction rate (Wang C.K. et al., 2015).

Diarrhea is a common side effect of chemotherapy with poorly understanding of mechanism. The absolute percentage of diarrhea caused by chemotherapy has yet to be accurately examined. Although diarrhea is a well-recognized side-effect of chemotherapy, little research has been conducted focusing on improving the treatment of chemotherapy-diarrhea (Wadler et al., 1998). Though the inhibiting DNA topoisomerase I of the tumor cells, irinotecan hydrochloride (CPT-11) is found to be a relatively new chemotherapeutic agent, which is used to treat a variety of solid tumors (Lenfers et al., 1999). SN-38 is an active form of CPT-11, which is converted to liver, and subsequently combined into an inactive, non-toxic SN-38 glucuronic acid. After that, bacterial β-glucuronidase can degrade SN-38 glucuronide to SN-38 and activate it, thus the toxicity and its side effect such as diarrhea is induced (Chen et al., 2013).

Hangeshashinto is a compound formula made by seven herbs and is commonly used in Japan to treat diarrhea and acute gastroenteritis (Hatakeyama et al., 2015). The effect of Hangeshashinto was to ease the enterohepatic circulation of SN-38. Baicalin, a compound from hangeshashinto, has been shown to inhibit the activity of β-glucuronidase as well as the synthesis of prostaglandin E2 (Kono et al., 2014). A clinical study involving 95 patients diagnosed with colorectal cancer showed that hangeshashinto was useful to allay the severity of diarrhea caused by CPT-11 in FOLFIRINOX therapy. Compared with placebo group, the patients who received hangeshashinto combined with chemotherapy have lower diarrhea occurrence obviously, which demonstrated that it had significant effect to alleviate the chemotherapy-induced diarrhea (Shindo and Koike, 2011).

Herba kummerowiae is a traditional Chinese herb whose functions mainly are heat-clearing, detoxicating, promoting urination and anti-diarrhea. It also has been used in the treatment of gastroenteritis, dysentery and urinary system infection (Wang W. et al., 2015). From animal experiment, the reduction of the loose stools rate and diarrhea index were caused by Herba kummerowiae and formula containing Herba kummerowia such as xiaoer xiesuting granule and montmorillonite powder (Liu et al., 2013).

## Hematological Toxicity

Hematological toxicity is one of the main reasons for tumor patients to stop chemotherapy. Because of these toxicities, the production of red blood cells (anemia), production of white blood cells (neutropenia or granulocytopenia), and production of platelets (thrombocytopenia) were decreased, which may threaten the patient’s life (Testart-Paillet et al., 2007). Myelosuppression causes the decrease of red blood cells, platelets and white cells. This is because the production of all blood cells is affected (Kambhampati et al., 2015).

A meta-analysis showed that chemotherapy combined with traditional herbal medicines is usually better than use chemotherapy alone on the pooled results of WBC count. RCTs to evaluate the hematological toxicity of cancer patients treated with radiotherapy or drug therapy or TCM combined with chemotherapy were reviewed and summarized in Table 2 (Teschke et al., 2015). The hematological toxicity was measured mainly according to World Health Organization (WHO) criteria (Table 2). Evidence from the RCTs showed that TCM can be used as an adjuvant to alleviate bone marrow suppression induced by chemotherapy or radiotherapy, especially to reduce grade III-IV toxicity. In the process of chemotherapy, Chinese herbal compound for toxifying kidney and spleen can increase WBC count and reduce the incidence of leukocyte reduction (Li et al., 2015).

**Table 2:** Clinical trials of using TCM combined with chemotherapy to decrease the hematological toxicities.

Number of patients		Type of cancer	CT regimen	Type of TCM	Outcomes	Modified Jadad scores	Reference
						
Treatment	Control						
30	30	Ovarian cancers	6	BASIC FORMULA	➀➂	3	(Chan et al., 2011)
30	30	Breast cancer	4	Shugan jianpi decoction	➀➁➂	4	(Chen H. et al., 2015)
30	30	Ovarian cancers	3	Yiliu decoction	➀➁	3	(Chen, 2012)
78	78	Throat cancer	4	Qingliulianghou decoction	➀➁➂	3	(Chen et al., 2012)
30	30	NSCLC	4	Yiqiyangyin decoction	➀➁➂	3	(Huang et al., 2011)
31	31	Colon cancer	4	Yiqiyangxue decoction	➀➁➂	3	(Li, 2015)
62	62	NSCLC	4	Fuzhengkangai decoction	➀➁➂	3	(Jun, 2008)
23	23	Ovarian cancers	4	Fuzheng quyu decoction	➀➁	3	(Liu, 2008)
61	61	Breast cancer	3	Wenshen Shengbai decoction	➀➁	3	(Lu et al., 2014)
30	30	NSCLC	3	Fuzhengxiaoyan decoction	➀➁➂	3	(Sun, 2011)
34	34	Breast cancer	4	Kangliuzengxiao decoction and feiyanning decoction	➀➁➂	2	(Wu, 2016)
58	58	NSCLC	5	Kangliuzengxiao decoction and feiyanning decoction	➀➁➂	4	(Xu et al., 2007)
62	62	NSCLC	4	Kangliuzengxiao decoction and feiyanning decoction	➀➁	3	(Xu et al., 2011)
63	63	NSCLC	4	Kangliuzengxiao decoction and feiyanning decoction	➀➁➂	3	(Zhu et al., 2011)


*➀Low WBC (white blood cell); ➁Granulocytopenia; ➂Thrombocytopenia; NSCLC, Non-small-cell lung cancer; TCM, Traditional Chinese Medicine.*

Elemene, an extract from the traditional Chinese medicinal herb Curcuma wenyujin, is a mixture of β-, γ-, and δ-elemene with β-elemene as the main components. β-elemene (1-methyl-1-vinyl-2,4-diisopropyl cyclohexane) is the plant’s main ingredient related to anti-tumor effect (Yao et al., 2008). A meta-analysis showed that β-elemene can increase treatment efficacy by improving survival, tumor response, and performance status and by reduction of the toxicity induced by chemotherapy for lung cancer, especially for NSCLC. The analysis involved 21 publications, all of which demonstrated that patients who treated with elemene-based combinations were more sensitive to chemotherapy than patients who received chemotherapy alone (Wang et al., 2012).

Shenqi Fuzheng injection (SQI) is an injection composed of *Codonopsis pilosula* (Franch) Nannf. and *Astragalus membranaceus* (Fisch.) Bunge and was approved by the State Food and Drug Administration of the People’s Republic of China (SFDA) in 1999 (Bai et al., 2003) as an adjuvant drug for lung cancer and gastric cancer for the use of tonifying and strengthening the body’s defense. Published trials have shown that SQI could improve tumor response and increase immunity indicators in cancer patients with treatment of chemotherapy (Dong et al., 2010). A meta-analysis showed that SQI combined with chemotherapy can improve the levels of NK, CD3+, CD4+, and CD8+ cells. Life quality improvement rate of combination treatment group was about twice of that in chemotherapy group [RR = 2.02, 95% CI (1.81, 2.26), *P* < 0.000 01]. Immune function test showed that combination treatment group was 3.2 (standard deviations) times greater than chemotherapy group [MD = 3.23, 95% CI (2.86, 3.60), *P* < 0.000 01] (Hao et al., 2015).

*Ganoderma lucidum* (Rokkaku-Reishi, RR), a traditional Japanese herb, has been used as a traditional supplement for human health. An animal study confirmed that it had immune-potentiating effects by activating T cells in long-term treatment (Kohguchi et al., 2004). Another animal study showed that the recovery of compared with control group, the count recovery of CD8+ and NK1.1+ cells in the spleen was increased in Ganoderma lucidum group. Cyclophosphamide treatment can result in the decrease of leukomonocyte in spleen and the abnormal splenocytes gain and *G. lucidum* can alleviate these side effects. These results suggested that the reduction of QOL caused by chemotherapy such as cyclophosphamide could be improved by ganoderma lucidum because of its beneficial effects (Nonaka et al., 2005).

Juzen-taiho-to (TJ-48) can alleviate the hematological toxicities induced by chemotherapy. From the animal and clinical trials, it is confirmed that TJ-48 has the extremely low toxicity (LD50 > 15 g/kg op murine), it has self-regulation and synergism in immunomodulation and immunoenhancement because it can stimulate hematopoietic impressions and produce interleukin with NK cells. Therefore, TJ-48 may be combined with many chemotherapeutic drugs to enhance the therapeutic effect of chemotherapeutic drugs and prolong the survival time. At the same time, it can also prevent or improve the side effects caused by chemotherapy, such as nausea and vomiting, bone marrow transplantation, immunosuppression and so on (Zee-Cheng, 1992). A clinical study involving 130 patients confirmed that TJ-48 can prevent bone marrow suppression induced by chemotherapy-treated breast. Combination of TJ-48 showed improvement in QOL of breast cancer patients compared with chemotherapy alone (Adachi and Watanabe, 1989). Another clinical study revealed that TJ-48 reduced the atrophy of the testis, thymus and spleen caused by mitomycin C (MMC) and had beneficial effects on leukopenia, anemia and body weight loss caused by MMC. Combination of TJ-48 can also suppressed the of BUN and creatinine caused by cisplatin (Iijima et al., 1989). What’s more, an animal study showed that TJ-48 can alleviate myelosuppression induced by chemotherapy, increase the WBT count and ratio of CD3+ and CD4+ and bone marrow cells (Ogawa et al., 2012).

LCS101 is a botanical compound formula being develop based on the natural products for the treatment of patients with solid cancers (Yaal-Hahoshen et al., 2011). The cytotoxicity of LCS101 is selective and targeted, which can cause the cell necrosis, but LCS101 has no adverse effect on normal cancer-free epithelial cells. In addition, LCS101 can reduce the damage to normal epithelial cells caused by chemotherapeutic drugs such as doxorubicin and 5-fu, and enhance their cytotoxicity to cancer cells (Cohen et al., 2015).

In Indian, there is a compound formula named Rasayanas, which is composed of a group of complex herbal drugs. The Rasayanas is widely used in a tradition system of medicine named Ayurveda to enhance the body health (Vayalil et al., 2002b). An animal study showed that Rasyanas were effective myeloprotectors in mice, and recovered the body and organ weight loss obviously affected by cyclophosphamide and radiation (Vayalil et al., 2002a). From an animal study with mice treated by cyclophosphamide and radiation, it is reported that Rasayanas can prevent the tissues from cytotoxic injury caused by reduction of serum levels and liver lipid peroxides and alkaline phosphatase related (Menon et al., 1997).

## Oral Mucositis

Oral mucositis is one of the most commonly complications of chemotherapy. After receiving the first chemotherapy cycle, about 18–40% of patients will have this symptom. The pain related to oral mucosal inflammation and elcosis is the main cause of oral mucositis. Oral mucositis affects the mouth hygiene, food intake and QOL (Chan et al., 2003). Patients report that mucositis is the most debilitating side effect of their head and neck cancer therapy. Feeding tube placement, hospitalization, and intensive supportive care may be required to deal with mucositis, which largely decrease the QOL of patients with cancer (Trotti et al., 2003).

EP2 signal is related to cell proliferation and metastasis in several kinds of cancer. There are several regulated ingredients of prostaglandin E2 in Hangeshashinto, a traditional compound formula. From a retrospective study, 14 patients with oral mucositis caused by gastric cancer chemotherapy received hangeshashinto treatment. The result showed that the risk of occurrence in the patients who have grade 1 oral mucositis is improved by hangeshashinto, though it was not observed to have effect on the reduction of incidence of oral mucositis whose grade ≥ 2 (Aoyama et al., 2014). Another clinical study involved patients with head and neck who had mucositis after receiving chemotherapy showed that hangeshashito can significantly improve the side effects caused by chemotherapy including cisplatin (Matsuda et al., 2015). In addition, serum albumin level was better maintained in the hangeshashinto combined with chemotherapy group than in the chemotherapy using only group.

Rhodiola algida is a Tibetan plant. It has been used to be a Chinese medicine for thousands of years. From nourishing spirit and promoting blood circulation to remove blood stasis, it can affect the immune system of human. It has been confirmed that it can increase the endurance, recover from fatigue and stimulate the nervous system (Li et al., 2009). A clinical study showed that Rhodiola algida can enhance the levels of IL-2, IL-4, granulocyte-macrophage colony-stimulating factor and the mRNA content of these cytokines and make the white blood cell levels return to normal range quickly to increase immunity and decrease the quantity of oral ulcerative mucositis of patients who receive the chemotherapy (Loo et al., 2010).

A cell study identified that hangeshashinto (HST), a traditional Japanese medicine, was effective for the treatment of oral mucositis. The enzyme immunoassay and liquid chromatography-tandem mass spectrometry (LC-MS/MS) revealed that inducible PGE2, PGD2, and PGF2α, metabolites of cyclooxygenase (COX) pathways were reduced by HST (10–300 micro/mL) without inducing cytotoxicity to the cells. The active ingredients of HST were identified by LC-MS/MS, among which 6-shogaol, 6-gingerol, wogonin, baicalein, baicalin, and berberine were shown to reduce PGE2 production (Hitomi et al., 2017).

In China and Japan, there is a well-known traditional herb medicine named Oren-gedoku-to (OGT). It is usually used to treat senile dementia in clinical therapies. A clinical study involving 40 patients with acute leukemia showed that OGT can significantly prevent mucositis caused by anticancer agents. Compared with patients in control group who received treatment with a gargle including sodium gualenate, the occurrence of stomatitis was 27.9% in the group received oren-gedoku-to, which was obviously lower. The occurrence of diarrhea in oren-gedoku-to group (9.3%) was also largely lower than control group (31.7%) (Yuki et al., 2003).

## Cardiotoxicity

Cardiotoxicity refers to the direct toxicity of the heart, which is divided into direct and indirect injury. The direct damage acts on the heart muscle, while the indirect damage is affected by the electrophysiological changes of heart sinus rhythm. Cardiotoxicity is not a common side effect of chemotherapy; however, reports of cardiac toxicity from the use of chemotherapeutic drugs have increased over the past decade. Symptoms of cardiac toxicity include arrhythmia, myocardial ischemia, diastolic disorders, myocardial infarction, angina pectoris, pericarditis, and heart failure. However, In the PubMed, there are no publication reported that the natural products combined with chemotherapy to decrease the cardiotoxicity induced by chemotherapy in clinical trial (Wu et al., 2015).

Five active components were found in Zhenqi FuZheng granule (ZQFZ) by liquid chromatography-mass spectrometry. Among them, hydroxytyrosine, neonyl ether, salidroside and cimetidine could inhibit the cardiotoxicity induced by DOX. Western blot results showed that the apoptosis-related Bax/bcl-2 protein was down-regulated under the action of these active substances. At the same time, the accumulation of reactive oxygen species (ROS) was reduced, so the side effects of cardiac toxicity were inhibited (Chen Y.Q. et al., 2015).

## Hepatotoxicity

Hepatotoxicity is one of the most important adverse drug reactions associated with antituberculosis chemotherapy (Ohkawa et al., 2002). Chemotherapy drugs can cause a variety of liver damage. Among them, platinum drugs such as cisplatin and oxaliplatin can greatly damage the ability of the sinusoidal liver and destroy the blood vessels that transport oxygen to the liver (Mikalauskas et al., 2011). The reason for hepatotoxicity is that they produce ROS from mitochondria in sinusoidal epithelial cells. These ROS increase cytokines and make normal hepatocytes more sensitive to apoptosis and cell damage (Kebieche et al., 2009). Some studies have shown that the level of CYP450 may be associated with level of hepatotoxicity, especially CYP2E1 and CYP4A11 proteins (Dambach et al., 2005). Hepatotoxicity limits the duration of treatment and dosage of drugs for cancer patients. However, there is no clear treatment for chemotherapy-induced hepatotoxicity. Some evidence shows that the use of antioxidants can reduce liver damage (McWhirter et al., 2013).

On the other hand, the benefits of herbal remain unproven and concern about adverse effects is leading to closer scrutiny of these products in clinical trial, while herbal hepatotoxicity has been recognized for many years, including steatosis, acute and chronic hepatitis, hepatic fibrosis, zonal or diffuse hepatic necrosis, bile duct injury, acute liver failure requiring liver transplantation and carcinogenesis (Table 3). At present, it is important to aware that Potential interactions between herbal medicines and conventional drugs may interfere with patient management.

**Table 3:** Some examples of causes of hepatotoxicity by Tradition Chinese Medicine (TCM).

Herbal medicine	Use	Components	Type of hepatic injury	Reference
TCM	Skin disease	Many	Liver injury	(Teschke, 2014)
TCM	Health tonic	Many	Vanishing bile duct syndrome	(Teschke, 2014)
TJ-9	Liver disease	Many	Acute and chronic hepatitis	(Danan and Teschke, 2016)
TJ-9	Tonic, biral hepatitis	*Scutellaria* and others	Fibrosis	(Teschke and Eickhoff, 2015)
Jin Bu Huan	Sedation	Lycopodium serratum	Acute and chronic hepatitis	(Benichou et al., 1993)
Chinese herbal tea	Health tonic	Compositae	VOD	(Teschke, 2014)
African remedy	Multiple uses	Atractylis gummifera	Diffuse hepatic necrosis	(Joy and Nair, 2008)
Bajiaolian	Multiple uses	Podophyllotoxin	Abnormal liver tests	(Xiong et al., 2016)
Ma-huang	Slimming aid	Ephedrine	Acute hepatitis	(Osama et al., 2017)
Prostata	Prostatism	Saw palmetto	Fibrosis	(Chen, 2009)
Sassafras	Herbal tea	Sassafras albidum	Hepatic carcinogenesis in animals	(Joy and Nair, 2008)
Margosa oil	Health tonic	Azadirachta indica	Reye’s syndrome	(Teschke, 2014)
European remedy	Gallstones	Chelidonium majus	Hepatitis	(Cui et al., 2017)
Chaparral leaf	Multiple uses	Larrea tridentata	Chronic hepatitis	(Chen and Wang, 2017)


*TJ-9: A kind of Chinese medicine used in China and Japan, which includes Sho-saiko-to and Dai-saiko-to; VOD, venoocclusive disease.*

HILI (herb induced liver injury) is a serious problem which restrict the use and development of natural herbal products (Jing and Teschke, 2018). Although the hepatotoxicity of herbal medicine is of great clinical and regulatory significance, the major problem that patients who have HILI seemingly face is the lack of rigorous causality assessment. This problem is best solved by using a hepatotoxicity specific CIOMS/RUCAM scale and estimation of unconscious re-exposure text (Frenzel and Teschke, 2016).

**FIGURE 3:**
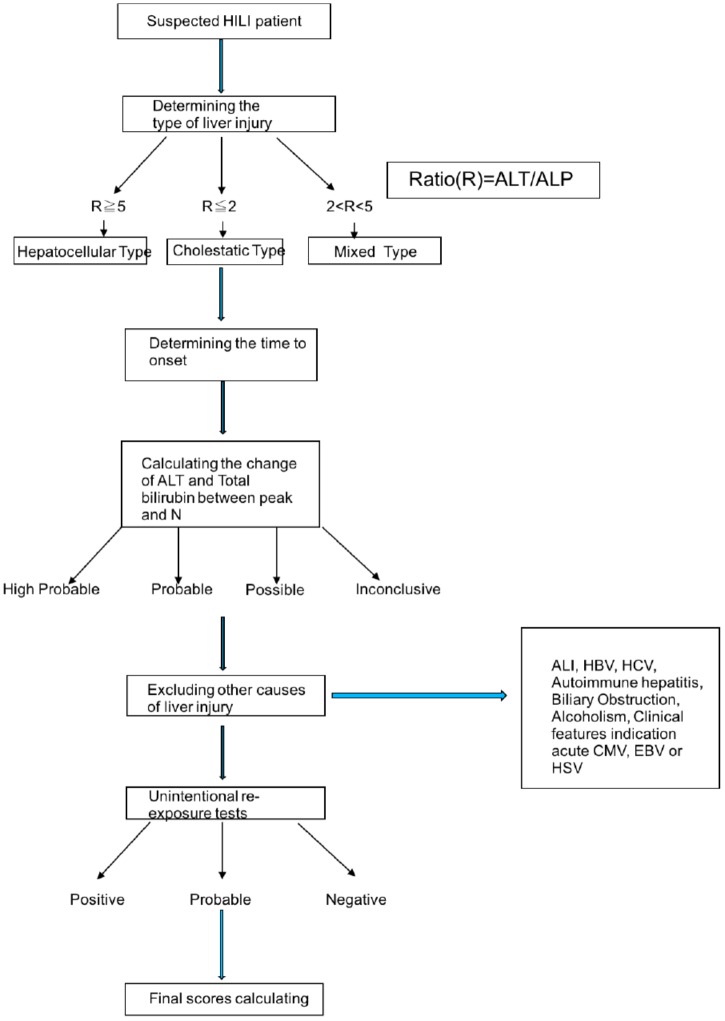
The procession of diagnosing HILI by RUCAM scale. ALT, alanine aminotransferase; ALP, alkaline phosphatase; HILI, herbal induced liver injury. HBV, hepatitis b virus; HCV, hepatitis c virus; CMV, cytomegalovirus; EBV, Epstein-Barr virus; HSV, herpes simplex virus.

RUCAM scale has well-defined evaluation criteria for hepatotoxicity, which includes all steps to diagnose the HILI (Figure 3). In the most HILI cases, ALT or ALP levels are considered the best criterion for diagnosis of HILI. *N*-value is the normal range of ALT and ALP. HILI is commonly considered diagnostic when the ALT value is over 5N or the ALP value is over 2N, or the ALT value is over 2N while the total bilirubin exceeds 2N. Meanwhile, it should be combined with the time between the herbal medicine and the onset of the disease. Other possible liver biochemical abnormalities such as hepatitis virus infection, alcoholism, fatty liver, autoimmunity should also be eliminated. The international consensus divides HILI into hepatocellular injury, cholestatic injury and cholestatic-hepatocellular injury (Teschke et al., 2014). The ratio (R) is the calculation of ALT/ALP, which is the expression of enzyme activity. In addition, because of the lack of biomarkers for all HILI cases, it is important to use the re-exposure test result as the gold standard to verify the diagnosis. Required data are baseline ALT levels before re-exposure and the re-exposure ALT levels. If the re-exposure test is positive, the HILI diagnosis can be confirmed (Teschke, 2014). However, re-exposure test can only be occurred unintentionally because of the high risk of severe liver injury and immorality of intentional re-exposure, so in the HILI cases, unintentional re-exposure is rarely recorded (Frenzel and Teschke, 2016).

For the suspected HILI cases, it is best to establish the causality for herbs in patients is to use RUCAM whose current version updated in 2016 (Danan and Teschke, 2016). A complication shows that numbers of international agencies and associated groups use RUCAM for HILI cases (Table 4; Teschke and Eickhoff, 2015). Compared with other causality tools, RUCAM has many advantages and is a wonderful tool to establish assess causality in HILI cases quantitatively. RUCAM represents a standardized and effective diagnostic approach for hepatotoxicity, which uses scores of key items to express the course of HILI. In case reports, the scores give final grade of causality for each suspected HILI patients (range from 14 to -3), which are highly probable, probable, possible, unlikely and excluded. RUCAM achieve the requirements that doctors have a great degree of confidence when they diagnose their patients who are suspected HILI, the results can be readily available in a few minutes (Danan and Teschke, 2016).

**Table 4:** Selective complication of agencies applying the RUCAM scale for causality assessment in suspected HILI.

Products	Country	Cases (n)	Reference
Various herbal TCM	Singapore	15	(Wai, 2006)
Black cohosh	Spain	44	(European Medicines Agency [EMA], 2007)
Herbs	Korea	13	(García-Cortés et al., 2008)
Various herbal TCM	Korea	159	(Kang et al., 2008)
Polygonum multiflorum	Korea	25	(Jung et al., 2011)
Various herbal TCM	Hong Kong	45	(Chau et al., 2011)
Natural herbal products	Various countries	73	(Bjornsson et al., 2012)
Various herbal TCM	Spain	15	(Robles-Diaz et al., 2015)
Kava	various countries	67	(Hardeman et al., 2008; Teschke et al., 2012)
Greater Celandine	various countries	21	(Teschke and Lebot, 2011)


Therefore, for the diagnose of HILI, it’s necessary to have an international sequential approach of case assessment. Use the updated RUCAM scale to have a clinical evaluation, followed by optional expert discussions based on RUCAM scores. This structured approach would help to improve the transparency of case data (Benichou et al., 1993).

## Nephrotoxicity

Nephrotoxicity refers to side effects of kidney damage. It is a broad term that includes all side effects associated with filtration, reabsorption and excretion. Chemotherapy-induced nephrotoxicity is one of the main factors limiting the time and dose of chemotherapy in cancer patients. Nephrotoxicity is especially severe when chemotherapy is combined with radiotherapy. Acute renal injury and hypomagnesemia are two common manifestations of nephrotoxicity. The study showed that 90% of cancer patients who received chemotherapy had nephrotoxic side effects (Joy and Nair, 2008). As for now, investigations about using natural products to decrease the nephrotoxicity induced by chemotherapy are mainly performed on animal experiments, and clinical trials are expected in the future.

Bu-zhong-yi-qi decoction (BZYQD, also called Zhong-Yi-Qi-Tangang, Bojungikki-tang, and Hochu-ekki-to) is a famous Chinese medicine prescription, which is extracted from eight kinds of Chinese herbal medicines and is widely used in Asia to improve digestibility. Animal experiments showed that 5-fu could lead to severe renal injury. BZYQD (1 or 2 g raw/kg/day, intragastrically) could reduce the apoptosis and necrosis of renal tubular epithelial cells and alleviate the side effects caused by nephrotoxicity through the antioxidant mechanism of BZYQD (1 or 2 g raw herb/kg/day, intragastrically). 5-fu treated mice showed morphological damage, increased urea- nitrogen and creatinine concentration, and decreased GSH-Px. BZYQD almost completely changed the renal function related indexes and antioxidant enzyme activity affected by 5-fu (Xiong et al., 2016).

Honey and royal jelly are daily health foods, and a clinical study has shown that acute renal damage caused by platinum chemotherapeutic drugs can be protected by the use of honey and royal jelly. Compared with the control group, serum levels of renal injury products were significantly decreased in cancer patients receiving honey and royal jelly capsules. At the same time, the changes of renal parameters were significantly lower than those of the control group after honey and royal jelly capsule therapy (*p* < 0.05) (Osama et al., 2017).

## Discussion and Conclusion

Chemotherapy is still one of the most commonly used cancer therapies which gained beneficial outcome to patients with tumor. Chemotherapeutic agents are rapidly discovered and developed by academia and industry, however, common adverse reaction and side effects are still difficult to overcome due tothe biological and chemical nature of the chemotherapeutics. Multiple component herbal products that have been ethno medically used for a thousand and hundreds of years have been proved their potential in reducing the side effects of chemotherapeutic agents, as summarized by our study. However, there are some problems remaining to be solved for facilitating the clinical application of these herbal products in the management of chemotherapy-induced side effects. First, the mechanism of action remains largely unclear. Although some experimental studies have used cellular model to explore some signaling transduction caused by herbal extract treatment, it is unlikely to be reproducible in animal and human; second, a great concern on herbal-drug interaction may be issued when the herbal products are considered to be used clinically, unfortunately, *in vivo* pharmacokinetic studies on both herbal products and the chemotherapeutic agents are scanty. Third, the uncertainty in composition of herbal extracts makes it difficult to gain a consistent efficacy in clinical application. Due to the nature and resource of herbal products, variance in quality between batch and batch products is often observed. This requires a more restricted way in quality control during production and manufacturing. Last but not least, the clinical trials related herbal products are not enough while the most experiments were still performed on the cell or animal platforms. More and more clinical trials with strictly followed protocols and highly standard design are important for illustrating the clinical efficacy and safeness of herbal products in the treatment of chemotherapy-induced side effects.

Compared with using conventional chemical drugs to decrease the side effects induced by chemotherapy, natural herbal medicines have many advantages. Because of the interactions of chemotherapy drugs and active ingredients in herbal medicines, it can have better effects than conventional chemical drugs. For example, CYP450 revulsive is used to be antidote in drug poisoning while it can reduce the efficacy of drugs at other time. Moreover, if the security of herbal medicines can be guaranteed, the natural products may help more patients to get treat, because natural products made by herbs are much cheaper than the conventional chemical drugs.

In conclusion, more and more evidence shows that compound drugs containing natural products can better reduce side effects caused by chemotherapy, thereby improving the QOL of cancer patients. However, since the compound is not a single natural product, the interaction and basic pharmacological effects of the active ingredients in the compounds need to be further studied in order to more clearly illustrate the mechanism of reducing the side effects of chemotherapy by natural compounds. In addition, rigorous clinical trials of drugs can provide reliable and decisive evidence, rather than just stay in cell and animal trials.

## Author Contributions

YF conceived the review. H-YT, SL, and FC collected and analyzed the data. BF and NW drafted the manuscript. All authors revised and commented on the paper and discussed the paper.

## Conflict of Interest Statement

The authors declare that the research was conducted in the absence of any commercial or financial relationships that could be construed as a potential conflict of interest.
